# Metafictive devices in children’s picturebooks and the development of children’s critical multimodal literacies

**DOI:** 10.1007/s44020-023-00032-8

**Published:** 2023-03-20

**Authors:** Carmel Turner, Georgina Barton, Stewart Riddle

**Affiliations:** 1 School of Education, Australian Catholic University, Brisbane, Australia; 2 School of Education, University of Southern Queensland, Springfield Central, Australia

**Keywords:** Children’s literature, Metafictive devices, Global issues, Learning and teaching, Critical literacy

## Abstract

High-quality children’s literature, including picturebooks, are important resources in the classroom for students to engage with complex and sometimes concerning contemporary issues. One strategy to involve students in learning about such issues is through the use of metafictive devices, which are literary stratagems that draw readers into knowing more about a topic and helping them understand and interpret them safely. In this paper, we analyse three selected texts that contain important and deliberate metafictive devices used by award-winning authors/illustrators. First, we share brief synopses of each book and then provide detailed analyses of the literary tools used by the authors, including how they support students’ meaning-making practices through language and image. Then we consider how these works were used in a primary school classroom to improve children’s critical multimodal literacies so that they became more discerning readers who could effectively cope and engage with complex and troubling world issues through literature.

## Introduction

Many children’s picturebooks deal with serious contemporary issues, including climate change (Axelrod et al., 2020), politics (Johnson, 2020; Sipe & Pantaleo, 2008), grief and loss (Wiseman, 2013), social justice (Dolan, 2014; Enriquez, 2014), and most recently, the global COVID-19 pandemic (Chen et al., 2020). Authors and illustrators often deal with complex social issues in children’s literature to support the development of children’s understanding and resilience (Murris, 2016). One literary strategy that can help young readers become more engaged with interactive narratives and tap into the deeper meanings of the text is the use of metafictive devices. For example, authors might tell a different story through words than what is told in the images (e.g. Anthony Browne’s book *Voice in the Park* on social class) or evoke empathy for particular characters or settings through the use of colour and emotive words (e.g. *How to Heal a Broken Wing* by Bob Graham and *The Red Tree* by Shaun Tan). The skills of authors and illustrators are often necessary to guide young readers’ engagement with sensitive topics that may be difficult for parents and carers to discuss with them.

In this paper, we present an analysis of three purposely selected texts that contain metafictive devices and address complex social issues. We provide brief synopses of each book and a detailed analyses of the literary tools used to emphasise specific meanings through both language and image. We then share data from a classroom ethnographic study in which the texts were introduced to students through a *Readers’ Circle* approach. Students explored the metafictive devices presented to them by encouraging them to use associated metalanguage to discuss the sensitive issues grappled with in each narrative. Finally, we consider some implications regarding how such discussions can improve young children’s critical literacies as readers so that they develop deeper understandings and resilience when learning about complex social issues.

## What are metafictive devices in children’s picturebooks?

Children’s picturebook authors and illustrators often use literary devices to draw readers into the topic at hand. Some of these devices are called metafictive devices, which are literary strategies that can combine different authorial or artistic elements within the text (Kümmerling-Meibauer, 2018). Examples of metafictive devices include using different approaches to narration such as the characters directly addressing the reader or having multiple narrators, referring to other texts within the narrative, and/or developing non-sequential or linear plots. It has been argued that metafictive devices allow children to infer deeper meanings represented in the language and/or images but, more importantly, encourage children to make greater connections to the text through their own lived experiences (Pantaleo, 2017, 2020; Zapata et al., 2017). Assisting children to connect personally and carefully with sensitive topics has led to further understanding and increased resilience (Dolan, 2013; Niu et al., 2021). Additionally, children can become more discerning readers by recognising that texts are deliberately constructed using artistic and literary devices for a purpose, which is often referred to as becoming critically literate (Farrar, 2017; So, 2016).

Defining characteristics of picturebooks containing metafictive devices have been catalogued by Sipe and McGuire (2006). They identified six inherent devices including:

1. The ‘blurring’ of boundaries in literary texts, particularly in relation to the relationship between author, narrator, and reader. This allows for a change in various hierarchies within a text through the juxtaposition of words. An example would be where a reader is carefully invited to ‘co-author’ or directly participate in the text. Here, the reader is no longer passive.
2. Subversion, or the change in status between the story and the ‘real’ outside world. The traditions of conventional stories may be destabilised as characters from a traditional story could suddenly act out of character or break the traditional frames between scenes of the story.
3. Intertextuality, or the layering of many different texts within the one text. This is a deliberate device used in picturebooks to create a pastiche of text, images, and, ultimately, meaning. This may include references to other texts or the insertion of artworks within the text.
4. Multiplicity of meanings within the story and the open-ended conclusions that differ from the ‘happily ever after’ endings present in the majority of children’s literature. Here, the same story could be told from many different perspectives but be set in the same place and time.
5. Playfulness, in which the use of semiotics engages the reader in a complex world of symbolism. This is where authors do not take themselves seriously and are semiotic and visual carnivals. The font style, shape, and size will vary within the one text, which will be placed in varying directions on the page and flow onto subsequent pages.
6. Self-referentiality, in which the reader is unable to view the text from ‘outside’ the story, but develops a relationship within the text. The assumption that the reader will become ‘lost in the book’ is cast aside.

According to Arizpe et al. (2008), there is potential for authors/illustrators to use these metafictive devices to encourage the deep reading of texts. We argue that educators can use these resources to provide further information about how these metafictive devices are used and the metalanguage related to each of them to support students’ literacy learning. We believe that a deeper understanding of an author’s intent through these metafictive devices can develop children’s critically literate voices and assist them in understanding sensitive societal issues, ultimately improving resilience and wellbeing.

## How metafictive devices might improve critical literacy

As highlighted above, an effective vehicle for developing students’ critical literacy can be deep engagement with picturebooks containing metafictive devices. Sipe (2008) argued that through stories we learn about other people, their cultures, and beliefs, which is vital for students as they begin to form their own worldviews as young citizens. Likewise, Moebius (1986) pointed out the importance of a story in the emergence of a worldview, which is formed by reading different perspectives and ways of knowing. The power of story means understanding narrative as not just entertainment, but as a vehicle that can inform and challenge as well as enrich students’ critical literacy development.

The term ‘critical literacy’ generally implies developing a reader’s ability to read with the understanding that (1) words and their interrelations can be used to offer different contexts, viewpoints, and positions from which readers can deconstruct and/or construct thoughts and ideas and (2) images (or other modes) can also stand alone, be part of a text or require interaction between image and text (Janks, 2014). A study to improve the critical literacy of children was carried out by Exley et al. (2014). It used three approaches to engage children in vulnerable schools with traditional fairy tales, which included exploring the generic structure and identity within texts, developing the children’s critical awareness of language through process drama, and engaging them in *Readers’ Circle* discussions. By using different strategies in the classroom that encouraged the deeper reading of texts, the study found that students developed a greater understanding and use of the metalanguage associated with textual and visual elements within the texts. The authors argued that supporting and encouraging children’s discussion of how the author/illustrator has presented information and what their intended meaning was is a powerful way to improve critical literacy. They also realised that both the opportunities and difficulties in this encouragement can sit alongside each other, alleviating concerns teachers might have regarding the implementation of more varied activities in their classrooms.

Rosenblatt (2004) argued that reading has many facets and is not only aesthetic or efferent but that readers need to read from a critical stance. Students need the skills to interrogate and build opinions about an author’s intent when they read about sensitive issues. It is also important that teachers support students in making these decisions. This can be made possible by giving students opportunities through quality multimodal literature such as picturebooks containing metafictive devices, to take an objective stance and develop a critically literate voice. As Janks (2019) argued, the skill of being critically literate enables students to interpret their current situation in which ‘fake news’ can infiltrate certain modes of communication (Turner & Griffin, 2019) and make sound judgements that may lead to societal change (Janks, 2020).

However, there are opportunities for reinvention and creativity and to experience storytelling in a way that presents students with a new worldview. Stories are told in a variety of ways in our multimodal world, and picturebooks allow educators to explore different modes of communication. Many young people are already challenging world leaders, voicing the younger generation’s concerns for the future (Riddle, 2022). However, these students need to be equipped with the ability to critically read these materials.

A study by Moses (2015) aimed to examine how bilingual students constructed meaning from the metafictive devices used in texts, with a focus on the images. Moses (2015) found that the narrative function of image varied including ‘image as access to meaning and content, image as prompt for discussion, image as a catalyst to seek access to written language, and image as a multimodal complement to written language’ (p. 82). Implications from this study highlighted the need for students to develop their metalanguage related to visual literacy and that the construction of multimodal texts assisted bilingual students to express meaning. Similarly, Papen (2020) implemented a strategy with years 5 and 6 children that involved picturebook discussions. They found that the children initially focused on the story rather than the deeper meanings related to the author/illustrator’s intent. They argued for a more ‘curious’ approach to reading picturebooks by explicitly teaching ‘how to read critically’.

Another study exploring the power of story and specifically visual literacy was undertaken by Ritone and Kurkjian (2018). They argued that picturebooks allow students to ‘increase awareness of visual and artistic aspects of texts and to convey to the viewer/reader’ (p. 31). They found that students positively engaged in the learning, including students who struggled with reading. The students particularly liked the online and technological aspects of the learning. This confirmed Serafini’s (2012b, 2015) observation that using picturebooks for their bimodal ensembles (i.e. language and image) to support literacy learning is beneficial. Additionally, Berger (1972) described an important defining characteristic of picturebooks containing metafictive devices, being the ‘gap’ between words and pictures filled by the reader engaging with the text. The use of the gap is important in these texts and can be viewed as the metafictive device—blurring of boundaries between reader and author, in which the reader is invited to co-author the text.

The authors and illustrators of these texts intentionally used this subtle disconnect to offer juxtaposing information between the illustrations and the words used in the text. For example, authors/illustrators carefully craft the words to be complementary to the images, but they may also be contradictory. This counterpoint can often create tension in the text. The use of counterpoint in picturebooks is stimulating because it elicits engaging responses from students (Nikolajeva & Scott, 2006).

While several studies have explored how different authors and illustrators use metafictive devices to convey meaning, limited information exists on how educators might draw on these devices to support students’ understanding of complex societal issues. Given the past few years have impacted greatly young children’s lives due to extended lockdowns as a result of COVID-19, serious natural disasters, and civil unrest such as those experienced in Ukraine and Afghanistan, we believe teachers can better support students through quality pedagogy involving picturebooks dealing with these issues. The next section of the paper describes the approaches taken to, first, analyse a selection of picturebooks that deal with sensitive issues as well as metafictive devices and, second, offer some ways in which teachers can use this information in the classroom in the hope that children’s understanding and resilience are improved.

## Research design

The study presented in this paper comes from a larger project that involved two phases. The first was to analyse ten picturebooks that dealt with contemporary sensitive issues and the second was to share these texts with young children in a primary school classroom to explore their engagement through critically literate discussion. Participants involved a class of children aged 7–8 years old (*n* = 23) and their teacher. The study aimed to explore how metafictive devices in picturebooks helped to support the development of teacher and student critical literacy and metalanguage. The study also aimed to assist students in being more aware of critical issues they might be facing personally.

The Australian Curriculum states that students are expected to read and comprehend above a basic literal level by becoming competent in analysing texts, drawing inferences, identifying an author’s point of view as well as their own, and critically evaluating meaning from a range of texts (ACARA, n.d.). According to Kress and van Leeuwen (2006), such competencies are critical if students are to fully engage with increasingly complex multimodal texts and the semiotic language embedded in these texts. Therefore, it is important that teachers provide opportunities for children to engage in picturebooks containing metafictive devices because these multimodal texts offer a richness of expression and artistic merit. They also contain context that reflects a rapidly changing and uncertain world at an appropriate level for students to begin forming their own critical and creative understandings of the world.

Once the texts were analysed, the first author worked in the classroom alongside the teachers and their students. A Readers’ Circle approach was used because this method allowed the development of students’ critical literacy skills to be observed (McLaughlin & de Voogd, 2004). Readers’ Circle is a dialogic form of literacy pedagogy, which is flexible, dynamic, adaptive, context-specific, and reveals students’ thinking as they critically analyse literary texts in the social context of their classroom. Conducted as an interactive group activity, the approach involved supporting students in reading and discussing each of the texts. During the Readers’ Circle activities, observation notes were taken and interviews with the teacher and students occurred afterward.

The texts shared in this paper were part of a larger selection of books included in a broader study undertaken by Turner (2020). For this paper, we selected three of 10 quality children’s picturebooks that address issues such as greed, social class and inequity, family conflict, and grief and loss. We analyse each text’s language and image for metafictive devices as identified by Sipe and McGuire (2006). Given space limitations, only three texts can be shared here, although their metafictive devices are representative of the larger text selection.

### The Short and Incredibly Happy Life of Riley

*The Short and Incredibly Happy Life of Riley*, written by Colin Thompson (2006) is about a happy rat named Riley who has everything he wants in life—a warm place to live, a home, and a loving family. The irony is that rats have a short life span compared with humans, yet humans are not as happy nor uncomplicated. Thompson compares people’s lives to that of a rat, and humans are not shown in a favourable light because of their constant quest to attain more and more materialistic possessions instead of being like Riley, who is satisfied with the basic requirements of life. Thus, the story highlights the greed of humans and their impact on our environment from such greed.

**Fig. 1:**
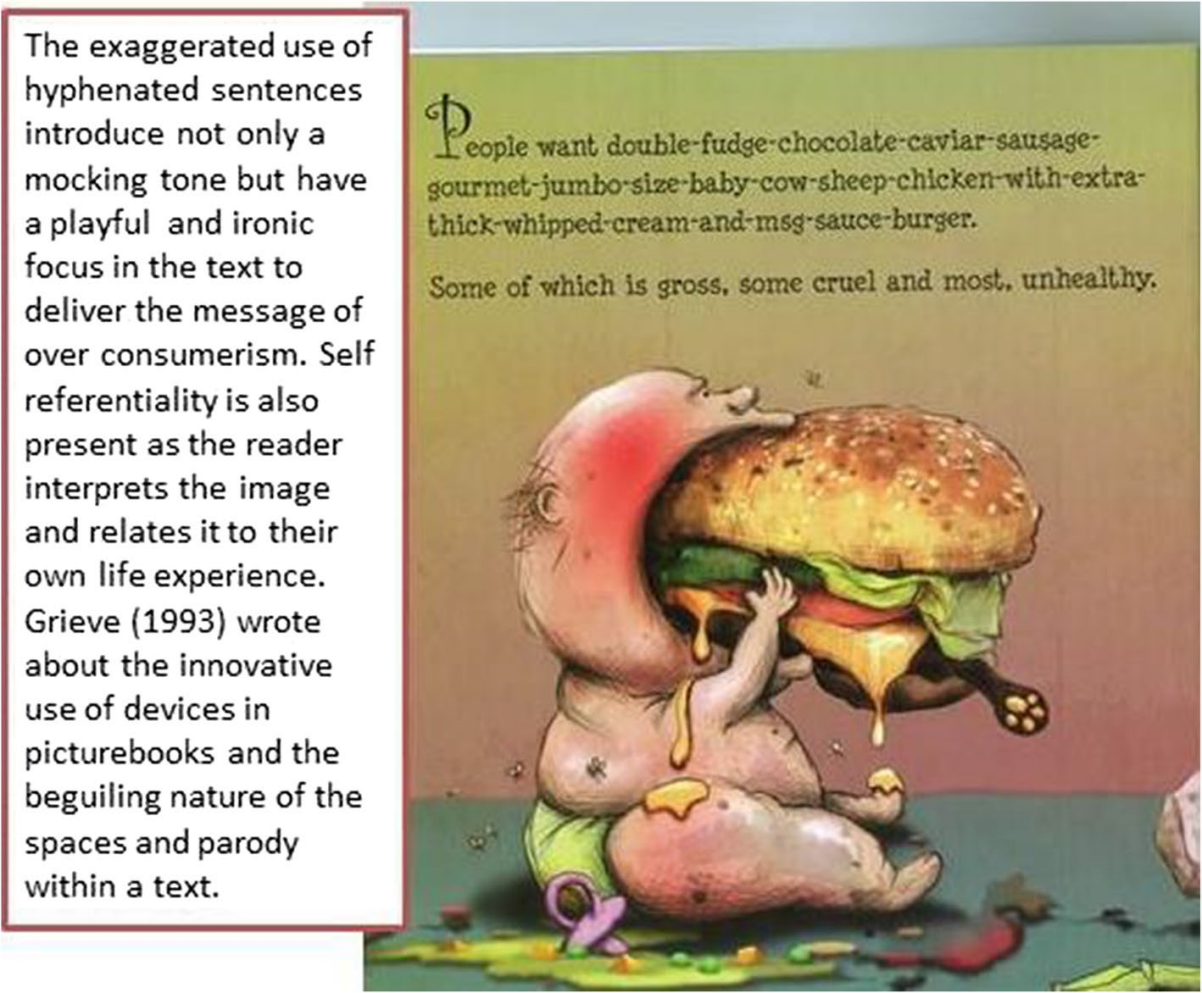
Metafictive devices in *The Short and Incredibly Happy Life of Riley*

This text is rich in metafictive devices. For example, Thompson uses a mocking tone to accentuate the fact that material possessions will not make you happy. He does this through the use of complex hyphenated sentences, the arrangement of words and pictures on the page, and the use of an elaborate font. This metafictive device, defined by Sipe and McGuire (2006) as playfulness, is demonstrated by the effect generated by the exaggerated hyphenation. When the text is read quickly, human greed becomes more obvious; for example, ‘people want double-fudge-chocolate-caviar-sausage-gourmet-jumbo-size-cow-sheep-chicken-with-extra-thick-whipped-cream-and-msg-burger’ (p. 9). This frenetic pace is contrasted with Riley’s needs: ‘All Riley wanted was a little stick with a pointy end to scratch the bit of his back he couldn’t reach himself’ (p. 11). While the text uses humour and irony, a message related to greed becomes apparent to the reader. Pantaleo (2014) further developed the definitions of Sipe and McGuire (2006) regarding metafictive devices by specifying the experimentation in the playful use of text (Fig. 1).

Intertextuality is another device that is used for effect in this text. There is a rich pastiche of famous paintings that have been graffitied. For example, van Gogh’s *Self-Portrait* (1889) has a bowler hat included as does da Vinci’s *Mona Lisa* (1503). These paintings, along with others, are used across a double-spread with hyphenated text that describes humans’ attempts to be ‘taller-shorter-thinner-here-but-much-bigger-there’ (p. 17). Munch’s painting, *The Scream*, is used on a page that states that if you compare your life to that of an animal, ‘you will only end up feeling depressed’ (p. 27).

The blurring of boundaries between author, narrator, and reader is also evident as Thompson adopts a conversational tone in his comparison of the lives of people and animals. The reader is drawn into the text in an almost conspiratorial mode, as if part of the mocking of humans’ obsession with acquisitions and a better life. Goldstone (2004) argued that these texts ‘prod and tease the reader’ as if asking them, ‘Do you understand what the story is about and what the author is trying to say?’ (p. 201).

Self-referentiality is another device used to great effect because the reader is familiar with many of the desires of the hapless humans in the text. The final page pushes home the meaning of the text in a simple sentence, which contrasts with the hyperbole that precedes: ‘And the answer is very simple, really, you just have to be happy with a lot less’ (p. 29). The illustration is of a man dressed very simply, eating an ice block, with a dog on a lead.

### The Tunnel

*The Tunnel*, written and illustrated by Anthony Browne (1989), focuses on the complexities of family relationships, including conflict and how to find resilience in such circumstances. The story unfolds around a brother and sister’s relationship that does not appear very strong because they have very different interests. The girl likes reading, and the boy playing soccer. These different interests are introduced to the reader in the endpapers. In the beginning, a book and a soccer ball are on separate pages; at the end, they are together on one page, indicating a shift in the sibling relationship. It is only on the final page that we learn the siblings’ names, Rose and Jack. The siblings are sent outside to play together by their mother. Reluctantly, they go to a disused allotment and follow their own pursuits. The brother notices a tunnel and tries to entice his sister to crawl through it with him. After a time, the brother does not return and his sister becomes increasingly worried. Finally, she crawls through the dark tunnel, worried that something has happened to her brother: ‘She waited and waited, but he did not come. She was close to tears. What could she do? She had to follow him into the tunnel’ (Browne, 1989, p. 11).

Browne employed a range of metafictive devices to tell this story of family redemption. The presence of intertextuality is present in the sister’s room with references to *Little Red Riding Hood*. As the sister runs through the forest, there are storybook characters carved in the tree trunks—a bear, a wolf, a gorilla, a pig, and various other creatures. These images reveal a text rich in images that need deciphering, with the reader wondering what they mean. Janks (2014) stated that texts position the reader to receive a message, and the more the reader engages, the greater the positioning by the author. Students need to realise that the author has this skill, and they need to decide whether to accept or reject the message being conveyed.

Complex visual images like those of *The Tunnel* present the reader with different pathways and possibilities about meaning. However, students need to be taught how to read not only the words but also the images; they also need to be encouraged to wonder why they are in the text and what information or point of view they represent. Nodelman (1988) agreed that readers need to understand not only words and pictures but what happens when they converge. The images and words either complement each other or are disparate. This means that readers should engage with both the text and images when reading. Words and pictures in a text complement each other and offer a richness of meaning that has to be decoded (Nodelman, 1988).

**Fig. 2:**
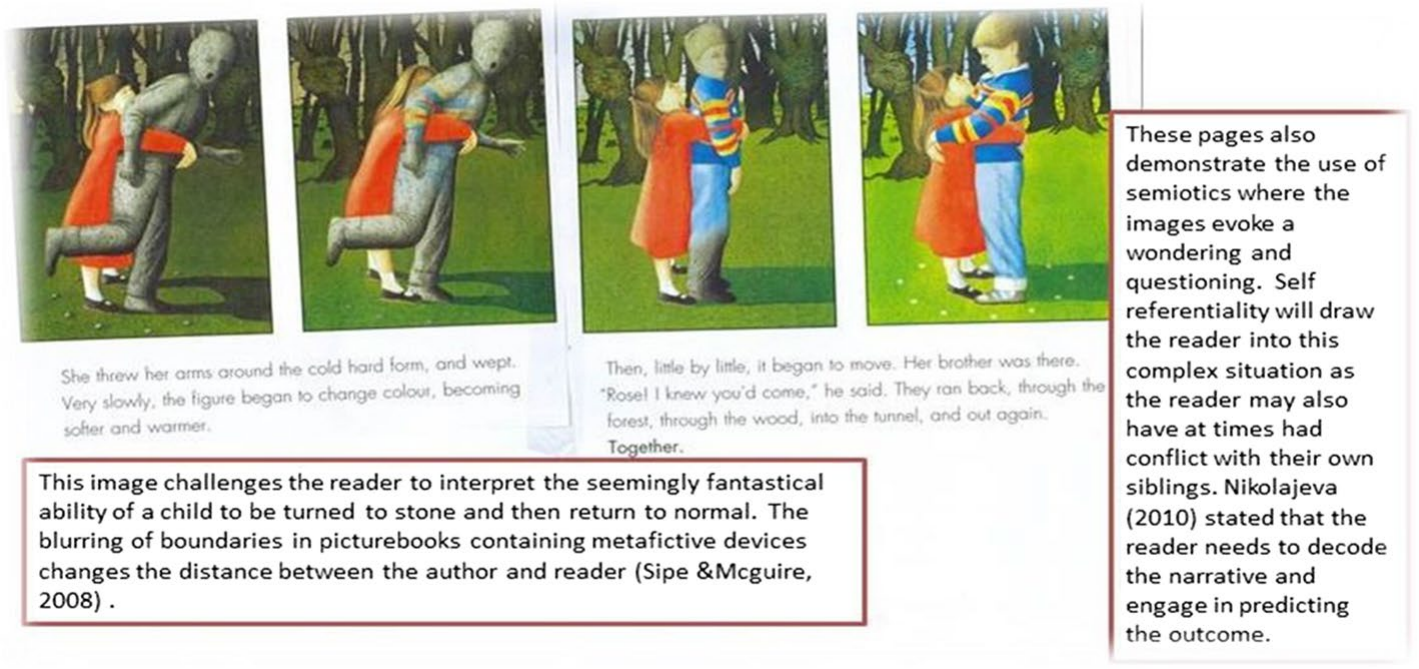
Metafictive devices in *The Tunnel*

The illustrations in these texts have an ‘ironic relationship to each other: The words tell us what the pictures do not show and the pictures tell us what the words do not tell’ (Nodelman, 1988, p. 222). This then offers the educator a unique opportunity to teach students the skills of being critically literate when presented with a text such as *The Tunnel*. What symbolism is represented by the brother turning to stone and the sister’s love returning him to him his former state? The symbolism of the brother turning to stone only to be released by his sister’s love (not the only possibility) reflects Sipe and McGuire’s (2006) definition referring to the multiplicity of meaning and open-ended aspect of these devices. The blurring of boundaries is also at play here, in which the reader is free to develop their own meaning of the text. The power of these devices is the opportunity for shared classroom discussion may unearth complex or literal meanings, depending on the students’ ability to decode the intersection of words and pictures (Fig. 2).

### The Heart and the Bottle

*The Heart and the Bottle*, written and illustrated by Oliver Jeffers (2010), is a thought-provoking and original picturebook that deals with the sudden absence of a father (or grandfather) from a young girl’s life and how she deals with the grief that accompanies such a loss. Again, this book introduces subtle ways in which children might try to be resilient in troubling times. This lavishly illustrated picturebook begins happily with a young girl and her father (or grandfather) walking with her outdoors and observing the world and inside sharing books and ideas about many topics such as the solar system.

Wonder is introduced, with the young girl revelling in the world around her. One day, she finds her father’s (or grandfather’s) chair empty; to cope with this reality, she places her heart in a bottle to keep it safe. The girl loses interest in the world and finds it awkward carrying her heart around her neck in a bottle. At the beach, the girl meets a younger girl who is still curious about her world. The girl does not know how to answer the younger girl’s questions, so she decides to take her heart out of the bottle, to restore her former self. However, she cannot work out how to do this. She tries many tactics, such as using a saw or a hammer. However, it is the younger girl who simply puts her hand in the bottle and removes the heart. So, the heart is put back where it belongs and the girl begins to heal. She regains her thirst for knowledge, sitting in the previously empty chair: ‘And the chair wasn’t so empty anymore. But the bottle was’ (pp. 30 − 31).

This text has a variety of illustrative styles that demonstrate Pantaleo’s (2014) focus on metafictive devices, notably the use of ‘typographic experimentation’ (p. 326) such as a simple double-page spread, multiple frames, and a complex collection of thought bubbles with images embedded in them. The illustrations of the girls are stylised in a simple sparse image. Illustrations with varied techniques, such as this text (especially the pages with detailed illustrations), often have a key detail to attract the reader’s eye and lead the reader to fill in the gaps in the text, a metafictive device defined by Sipe and McGuire (2006), in which the reader is invited to co-author the story (Nikolajeva & Scott, 2006).

**Fig. 3:**
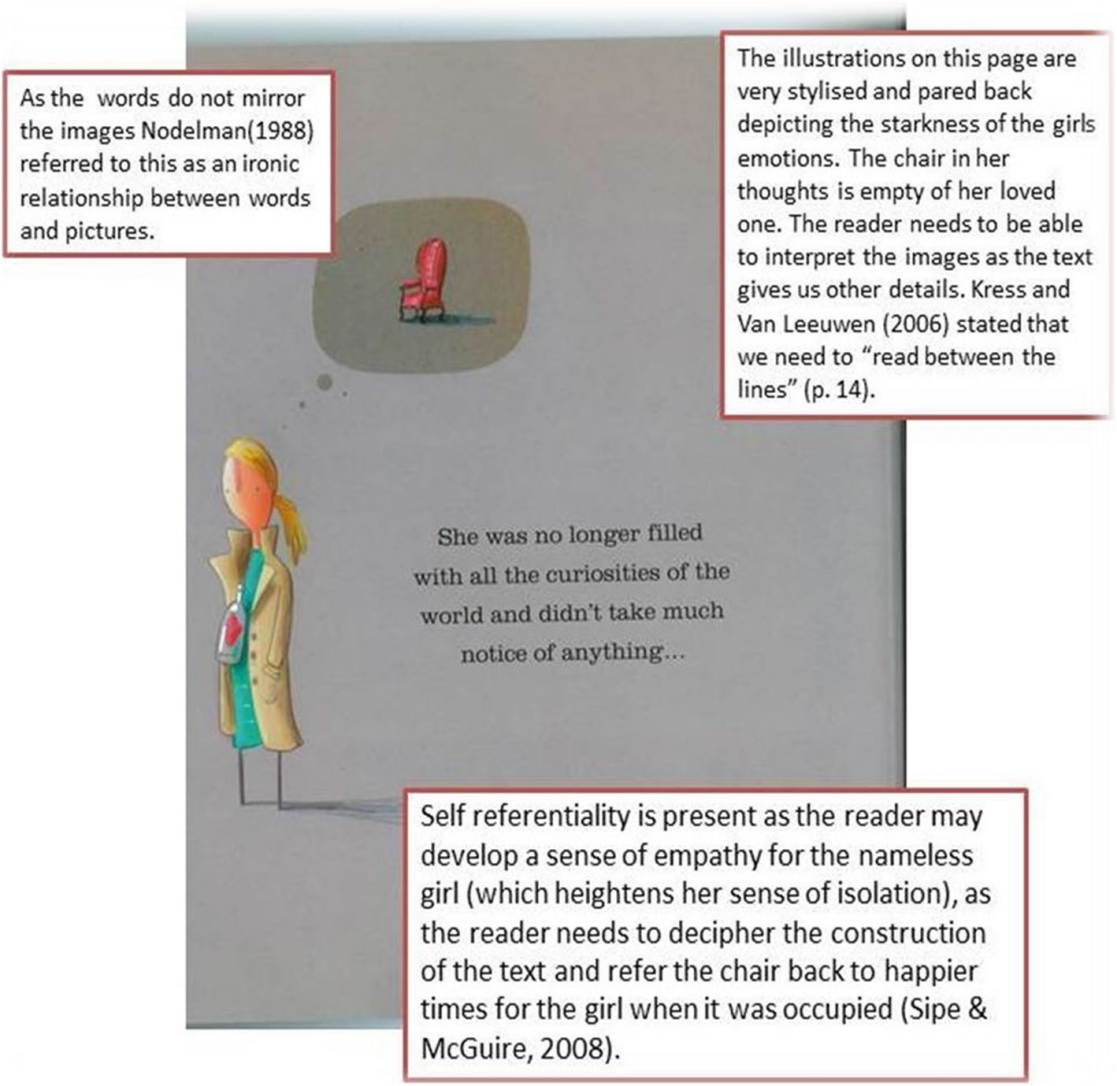
Metafictive devices in *The Heart and the Bottle*

Picturebooks can develop empathy in readers when the characters experience difficult situations (as in *The Heart and the Bottle*). The reader engaging with this text will need the ability to go beyond the literal to discover the message that the text carries. While it should never be forgotten that these picturebooks are written for pleasure, they do offer excellent opportunities for the development of a critical stance in uncovering their meaning. The recognition of the metafictive devices and the development of a metalanguage by the reader will assist in this development. The illustrator has used a specific visual element—framing—to draw the eye of the reader in to discover important information and provoke a feeling of wonder at the discoveries the girl has made. When she is alone and grappling with a broken heart, the page border is the frame, which conveys a feeling of being alone and lost. Smith (2009) observed that writers use the construction of a text to often disrupt the narrative and change what the reader was expecting. This text varies the use of framing to amplify the emotions of the story (Fig. 3).

Because picturebooks carry the bulk of their message in pictures, Jeffers (2010) has incorporated many metafictive elements into this medium as illustrated above. Sipe and McGuire (2006) refered to self-referentiality, in which the reader can no longer view the text as just a text but rather is drawn into engagement with the text. This excerpt from the text is sparse in detail but evocative of the absences presented. The multiple pathways into the text are also highlighted as a metafictive device used in picturebooks. Pantaleo (2014) argued that there are also gaps and silences within the text that provoke and raise questions as to the absence of the male figure within the text. The illustrations dominate the text, with varying arrangements and points of salience on the page. Painter et al. (2010) noted that children need to develop their literacy skills with a semiotic object that is not the voice of an adult. Thus, the images are very important in establishing the relationship between the reader and the text. The images can develop the tone of the text (Painter et al., 2010); Jeffers has managed to sensitively represent a serious topic through an illustrative style that is stylised, and not realistic. The changing of the focalisation of the images within the text moves the story along and draws the reader’s attention to important information that the text is conveying (Painter et al., 2010). The girl is depicted as a small figure against a larger landscape, which emphasises her loneliness and self-isolation. This contrasts with the beginning of the text, which is filled with excitement and wonder.

## Influence of the knowledge of metafictive devices on students’ critical literacy

Once the books were introduced and read to the students, they participated in a range of literacy learning activities related to the prose. Framed around Sipe and McGuire’s (2006) six inherent devices, in-depth conversations with the students occurred. For example, when exploring *The Tunnel* by Anthony Browne, the students demonstrated their growing awareness of the devices used by the author to convey a strong message related to how to deal with conflict (Box 1):

Box 1 Interaction 1, *The Tunnel*

**Table:** 

Teacher** What do you think about this illustration?**
Student 1 1: There’s lots of information and there’s lots of old pictures of this story and there’s a lot of paddocks.
**Teacher Yes, and there’s a lot of what?**
Student 1: There’s lots of the intertextuality
**Teacher Yes.**
Student 1: Because there’s pictures of stories in here.

Intertextuality had now become part of the conversation when discussing the messages in the texts and how the author told us the story. Luke and Freebody (1997) emphasised the importance of the social practices that readers engage in when making meaning of the text as well as readers’ abilities to analyse and critique texts, knowing that all texts reflect an author’s and illustrator’s intention (Luke & Freebody, 1997). The social interaction within the class was a strength that came through in this study; the students felt empowered by using the metalanguage associated with the metafictive devices as they developed their critical stance by evaluating the effectiveness of the authors’ use of devices. Further, responses from the students involved in this research reinforce the intended message from Jeffers, which deals with the confronting topic of an absent loved one. The responses also convey the tone and relationship of the text to the reader as indicated by Painter et al. (2010) (Box 2).

Box 2 Interaction 2, *The Heart and the Bottle*

**Table:** 

Student:1 It’s a story.
**Teacher: It’s a story and what is the author trying to tell us? Why would you put your heart away somewhere?**
Student: Because she’s really upset.
Student:2 When the girl didn’t realise her dad was there anymore when she took the heart out, she felt sad and lonely and she didn’t want anyone to come near her heart.
**Teacher: What do you think the story was about?**
Student:3 You can’t be the same without your love in your heart.
**Teacher: Why do you think you’d, in your head, say, ‘I’m putting my heart away, I don’t want it anymore.’?**
Student:4 She put it away because she was lonely and she was sad and she didn’t want anyone to touch her heart.
**Teacher: Yes. What else do you think?**
Student:5 Her heart might’ve been broken and she didn’t want it to break anymore so she wanted to put it in a safe place.
Student:6 Everyone has different feelings.
**Teacher: Excellent. Any other messages? What message did you get?**
Student: You have to move on.
Student: Just because your heart’s broken doesn’t mean you have to take all the love out of your heart.
**Teacher: Yes, what a wonderful answer.**
Student: Don’t hide your heart. Show it and get over it and make new friends.
Student: You won’t always feel alone and in the book, she had her heart in her body but the bottle was empty. It felt lonely.

Janks (2019) importantly argued that to critically understand a text the reader needs to engage at a level of understanding about how they are being positioned by the author and the intended message. The students in this study moved from a literal acceptance of a text to a deeper response as they recognised the voice of the author and the devices used by authors to convey their message. In relation to *The Short and Incredibly Happy Life of Riley*, the students discussed the author’s intent (Box 3).

Box 3 Discussion about author’s intent

**Table:** 

Teacher: Does anyone know what Colin Thompson’s message is? Student: You don’t need that many things to live and you can have less things and, if you have less things, you can live [happier] because you don’t need that many things to live.
Student: That you don’t need to change anything in your life. You just need to be happy.
Student: Be happy with what you’ve got and not sad with what you don’t have.

When asked whether the illustrations were necessary, they agreed they were because they helped to tell the story (although their reading behaviour did not demonstrate an interrogation of the illustrations at this early stage because they only read the words and quickly turned to the next section of text in the book). At this stage, the students were unaware of the power of authors to manoeuvre a reader into accepting a point of view. When a critical stance is enacted while reading, students have a heightened awareness of the devices used by the author and the knowledge that they as readers bring to a text. This critical stance is reflected in the Four Resources Model, in the role of text analysist. Luke and Freebody (1999) stressed that teaching literacy is about ‘institutional shaping of social practices and cultural resources, about inducting successive generations into particular cultural, normative ways of handling texts’ (p. 2). This was a critical point in this study because the students had not yet been exposed to this practice, as demonstrated by their lack of awareness of the whole text—that it was not just words with some decorative features but a multimodal text with two forms of communication. Initially, the ability to make meaning from this text was not strongly demonstrated by the students, who did not engage with the illustrations that are ever-present in their multimodal world, and in this case, literature (Unsworth et al., 2019). In *The Tunnel*, which focuses on sibling relationships, the student responses to a perplexing image of the brother turned to stone elicited this response:

**Table:** 

**Teacher She finds him looking like stone and then something happens – what happens?**
Student 1: She hugs her brother and –
**Q: And -?**
Student 1: And he turned back to normal again.
**Teacher: Well what does that tell you? What do you think that means?**
Student 2: The power of love changed him back to normal.

## Conclusion

In today’s world, young children face more complex social issues than ever before. The past two to three years is a case in point. All children across the world experience extended lockdowns due to the global COVID-19 pandemic and were consequently schooled at home. They may have also experienced issues related to a greater cost of living and even homelessness. In Australia, there are an increasing number of catastrophic natural disasters such as floods and bushfires as a result of climate change, which affect many children’s lives. In addition, children are often exposed to civil unrest and other world conflicts.

The purpose of this paper was to provide a detailed analysis of the metafictive devices in selected picturebooks that deal with such complex issues. Books addressing human greed, familial conflict, and grief and loss were shared in the hope that teachers and students may develop their knowledge and understanding of the power of story in (a) exploring complex issues that occur in people’s lives and (b) how people might build resilience and knowledge to overcome such challenges.

Professional authors and/or illustrators carefully weave words and image to create a beautiful narrative, usually for entertainment; however, they also assess deep issues related to being human so that children, parents/carers and educators, and other readers can consider how their own lives might relate and/or how they might deal with sensitive topics (Riddle, 2022). While it should always be acknowledged that these texts are written for the pleasure of the reader, to fully receive the intended message, a critical stance is essential. These complex texts offer a wonderful opportunity for educators to immerse students in quality literature, enjoyment of the narrative, and exploration of the illustrations while discussing the devices used by authors to convey their messages. Pantaleo (2014) argued that such texts have the potential to give students a medium through which to become more involved in exploring the text and cultivating the role of co-author by eliciting meaning from the text and filling in the gaps.

The adoption of a more critical stance (Luke & Woods, 2009) gives students the opportunity to connect their literacy skills to their own lives. In relation to reading and viewing, these skills can be developed and applied to the engagement with picturebooks containing metafictive devices; such books can contain pertinent messages about society and its values that are relevant to the students. Pantaleo (2014) argued that just because students live in a multimodal world does not necessarily mean that they are visually literate. The selected picturebooks in this paper demonstrate that through the devices used by the authors, important messages and the use of semiotics that give clues to unlock the deeper content of the text (Goldstone, 2004) are evident. By exploring the content and metafictive devices in the picturebooks in this study, it was evident that they offer a wonderful opportunity for students to explore the texts. Importantly, they also allow students to acquire a metalanguage, express their findings to peers, construct a shared understanding of the texts, and shape their stance about the text.

## References

[CR1] Anstey, M., & Bull, G. (2000). *Reading the visual*. Cengage.

[CR2] Arizpe E, Styles M, Cowan K, Mallouri L, Wolpert MA, Sipe LR, Pantaleo SJ (2008). The voices behind the pictures: Children responding to postmodern picturebooks. Postmodern picturebooks: Play, parody, and self-referentiality.

[CR3] Axelrod YD, Ives D, Weaver R (2020). We are all learning about climate change: Teaching with picture books to engage teachers and students. Occasional Paper Series.

[CR4] Benjamin, W. (2010). *The work of art in the age of mechanical reproduction.* https://www.marxists.org/reference/subject/philosophy/works/ge/benjamin.htm

[CR5] Berger J (1972). Ways of seeing.

[CR6] Browne, A. (1989). *The tunnel* (A. Browne, Illus.). Walker.

[CR7] Chen S-W, Moruzi K, Venzo P (2020). 26 May).

[CR8] Crawley SA (2017). Be who you are: Exploring representations of transgender children in picturebooks. Journal of Children's Literature.

[CR9] Dolan, A. (2013). Making development issues accessible through picturebooks. In *Meeting the Challenges of a Globalised World*&nbsp;(p. 127).

[CR10] Dolan, A. M. (2014). *You, me and diversity: Picturebooks for teaching development and intercultural education*. Trentham Books.

[CR11] Enriquez G (2014). Critiquing social justice picturebooks: Teachers’ critical literacy reader responses. New England Reading Association Journal.

[CR12] Evans, J. (Ed.). (2015). *Challenging and controversial picturebooks: Creative and critical responses to visual texts*. Routledge.

[CR13] Exley, B., Woods, A., & Dooley, K. (2014). Thinking critically in the land of princesses and giants: The affordances and challenges of critical approaches in the early years. In J. Zacher Pandya & J. Avila (Eds.), *Moving Critical Literacies Forward: A New Look at Praxis Across Contexts,* (pp. 59–70)*.* Routledge*.*

[CR14] Farrar, J. (2017). *‘I didn't know they did books like this!’ An inquiry into the literacy practices of young children and their parents using metafictive picturebooks* (Doctoral dissertation, University of Glasgow).

[CR15] Freebody P, Luke A (1990). Literacies, programs and debates in cultural context. Prospect: An Australian Journal of TESOL.

[CR16] Freire, P. (1970). *Pedagogy of the oppressed*. Bloomsbury.

[CR17] Goldstone B (2004). The postmodern picturebook: A new subgenre. Language Arts.

[CR18] Janks H (2014). Literacy and power.

[CR19] Janks H (2019). Critical literacy and the importance of reading with and against a text. Journal of Adolescent and Adult Literacy.

[CR20] Janks H (2020). Critical literacy in action: Difference as a force for positive change. Journal of Adolescent and Adult Literacy.

[CR21] Jeffers, O. (2010). *The heart and the bottle* (O. Jeffers, Illus.). HarperCollins.

[CR22] Johnson, K. A. (2020). *Collaborative critical practice: Designing a children’s picturebook with resettled refugees.* (Unpublished thesis). Texas State University.

[CR23] Kress, G., & van Leeuwen, T. (2006). *Reading images: The grammar of visual design* (2nd ed.). Routledge.

[CR24] Kümmerling-Meibauer, B. (Ed). (2018). *The Routledge companion to picturebooks*. Routledge.

[CR25] Lankshear C (2001). Literacy for sustainable development in the age of information. Linguistics and Education.

[CR26] Luke A, Freebody P, Muspratt S, Luke A, Freebody P (1997). Shaping the social practices of reading. Constructing critical literacies.

[CR27] Luke A, Freebody P (1999). A map of possible practices: Further notes on the four resources model. Practically Primary.

[CR28] Luke A, Woods A (2009). Critical literacies in schools: A primer. Voices from the Middle.

[CR29] Lysaker J, Sedberry T (2015). Reading difference: Picture book retellings as contexts for exploring personal meanings of race and culture. Literacy.

[CR30] McKinlay, M. (2011). *No bears* (L. Rudge, Illus.) Walker Books.

[CR31] McLaughlin, M., & DeVoogd, G. (2004). Critical literacy as comprehension: Expanding reader response. *Journal of Adolescent and Adult Literacy, 48*(1), 52–62.

[CR32] Moebius W (1986). Introduction to picturebook codes. Words and Image.

[CR33] Moses L (2015). The role(s) of image for young bilinguals reading multimodal informational texts. Language and Literacy.

[CR34] Murris, K. (2016). *The posthuman child: Educational transformation through philosophy with picturebooks*. Routledge.

[CR35] New London Group (1996). Pedagogy of multiliteracies: Designing social futures. Harvard’s Educational Review.

[CR36] Nikolajeva M, Scott C (2006). How picturebooks work.

[CR37] Niu W, Cheng L, Xu W, Zhang Q, Zhang X (2021). Improving resilience of a child with ADHD: A context specific intervention program through dialogic and guided reading. International Journal of Disability, Development and Education.

[CR38] Nodelman, P. (1988). *Words about pictures: The narrative art of children’s picturebooks*. University of Georgia Press.

[CR39] Painter, C., Martin, J., & Unsworth, L. (2010). *Reading visual narratives image analysis of children’s picturebooks.* Equinox.

[CR40] Pantaleo S (2005). Young children engage with the metafictive in picturebooks. Australian Journal of Language and Literacy.

[CR41] Pantaleo S, Evans J (2009). Exploring children’s responses to the postmodern picturebook: Who’s afraid of the big bad book?. Talking Beyond the Page: Reading and Responding to Picturebooks.

[CR42] Pantaleo S (2014). The metafictive nature of postmodern picturebooks. The Reading Teacher.

[CR43] Pantaleo S (2017). Critical thinking and young children’s exploration of picturebook artwork. Language and Education.

[CR44] Pantaleo S (2020). Slow looking: ‘Reading picturebooks takes time’. Literacy.

[CR45] Papen U (2020). Using picture books to develop critical visual literacy in primary schools: Challenges of a dialogic approach. Literacy.

[CR46] Riddle, S. (2022). Schooling for democracy in a time of global crisis: Towards a more caring, sustainable and inclusive future. *Routledge*.

[CR47] Ritone J, Kurkjian C (2018). Breaking the fourth wall: Using postmodern picturebooks to teach visual literacy in middle school. New England Reading Association Journal.

[CR48] Rosenblatt, L. M. (2004). The transactional theory of reading and writing: Theoretical models and processes of reading. *International Reading Association*.

[CR49] Serafini F (2012). Expanding the four resources model: Reading visual and multi-modal texts. Pedagogies: An International Journal.

[CR50] Serafini F (2012). Design elements of picturebooks: Interpreting visual images and design elements of contemporary picturebooks. Connecticut Reading Association Journal.

[CR51] Serafini F (2015). Paths to interpretation: Developing students’ interpretive repertoires. Language and Literacy.

[CR52] Serafini, F., & Reid, S. F. (2022). Analyzing picturebooks: Semiotic, literary, and artistic frameworks. *Visual Communication*, 14703572211069623.

[CR53] Sipe, L. (2008). *Storytime: Young children’s literary understanding in the classroom.* Teachers College Press.

[CR54] Sipe L, McGuire C, Lehr S (2006). The stinky cheese man and other postmodern tales for children. Shattering the looking glass: Challenge risk and controversy in children’s literature.

[CR55] Sipe LR, Pantaleo S (2008). Postmodern picturebooks: Play, parody, and self-referentiality.

[CR56] Smith V, Evans J (2009). Making and breaking frames: Crossing the borders of expectation in picturebooks. Talking Beyond the Page: Reading and Responding to Picturebooks.

[CR57] So JK (2016). Opening up spaces for early critical literacy: Korean kindergarteners exploring diversity through multicultural picture books. Australian Journal of Language and Literacy.

[CR58] Thompson, C. (2006). *The short and incredibly happy life of Riley* (A. Lissiat, Illus.). Hachette.

[CR59] Turner J, Griffin A (2019). Language and social change: A dialogue with Hilary Janks about critical literacy in a ‘post-truth’ world. Language Arts.

[CR60] Unsworth, L., Cope, J., & Nicolls, L. (2019). Multimodal literacy and large-scale literacy tests: Curriculum relevance and responsibility. *Australian Journal of Language and Literacy, 42*(2), 128–139.

[CR61] Wiesner, D. (2001). *The three pigs* (D. Wiesner, Illus.). Anderson.

[CR62] Wiseman AM (2013). Summer’s end and sad goodbyes: Children’s picturebooks about death and dying. Children’s Literature in Education.

[CR63] Zapata A, Fugit M, Moss D (2017). Awakening socially just mindsets through visual thinking strategies and diverse picturebooks. Journal of Children’s Literature.

